# Supplementary material for: CO Electroreduction Mechanism on Single-Atom Zn (101) Surfaces: Pathway to C2 Products
Source: Molecules. 2023 Jun 7;28(12):4606. doi: 10.3390/molecules28124606 (PMC10301100; doi:10.3390/molecules28124606)
Supplement: Supplementary file 1 [file molecules-28-04606-s001.zip › molecules-2433181-supplementary.pdf]

Supplementary Materials

CO Electroreduction Mechanism on Single-Atom Zn (101) Surfaces: Pathway to C2 Products

Yixin Wang [1,†], Ming Zheng [1,†], Xin Zhou [1,*], Qingjiang Pan [2] and Mingxia Li [2,*]

[1] MIIT Key Laboratory of Critical Materials Technology for New Energy Conversion and Storage, School of Chemistry and Chemical Engineering, Harbin Institute of Technology, Harbin 150001, China; 21s025052@stu.hit.edu.cn (Y.W.); 19b925114@stu.hit.edu.cn (M.Z.)

[2] Key Laboratory of Functional Inorganic Material Chemistry, Ministry of Education of the People's Republic of China, School of Chemistry and Materials Science, Heilongjiang University, Harbin 150080, China; panqingjiang@hlju.edu.cn

* Correspondence: zhoux@hit.edu.cn (X.Z.); limingxia@hlju.edu.cn (M.L.)

† These authors contributed equally to this work.

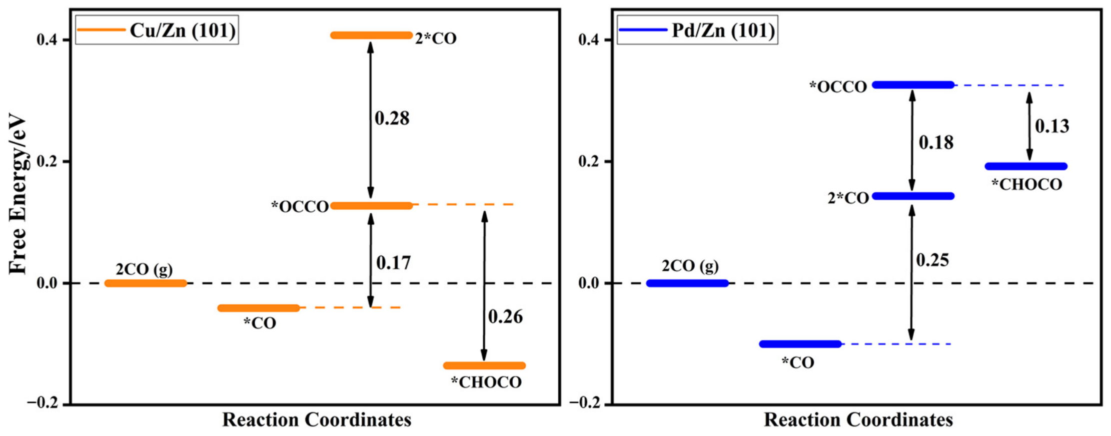

Figure S1. The CO dimerization mechanism of (a) Cu/Zn (101) and (b) Pd/Zn (101).

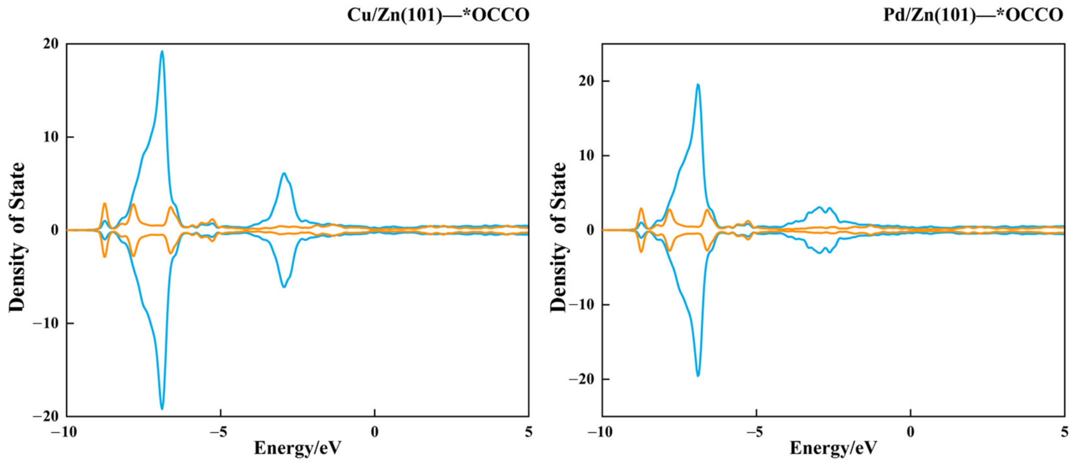

Figure S2. The DOS diagrams of Cu/Zn (101) and Pd/Zn (101) dimers with *OCCO, blue represents the DOS diagrams of substrate atoms bound with *OCCO, and orange represents the DOS diagrams of *OCCO.

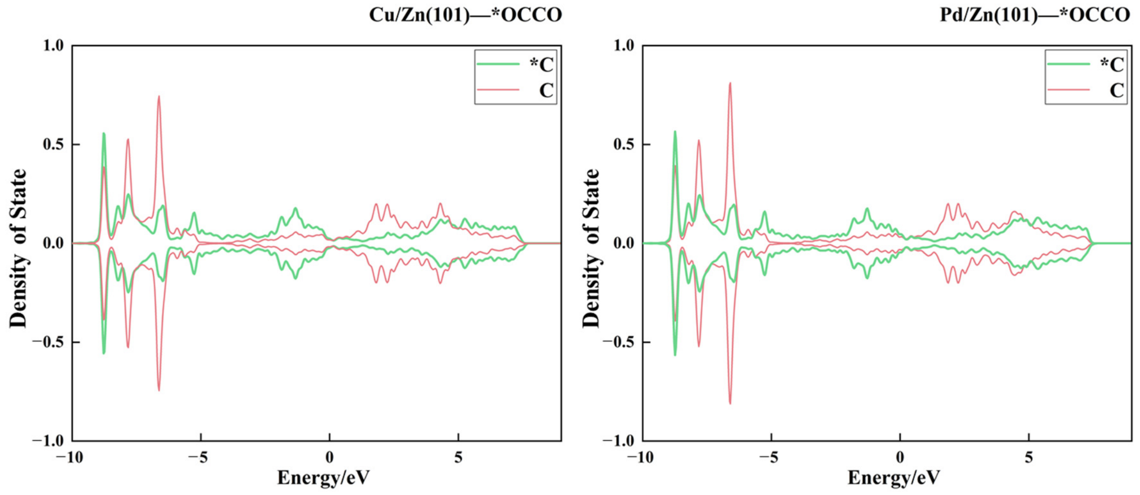

Figure S3. *The DOS diagram of the C-C bond in the OCCO intermediate, with green indicating the DOS of the carbon atom bound to the surface, red indicating the DOS of another carbon atom.

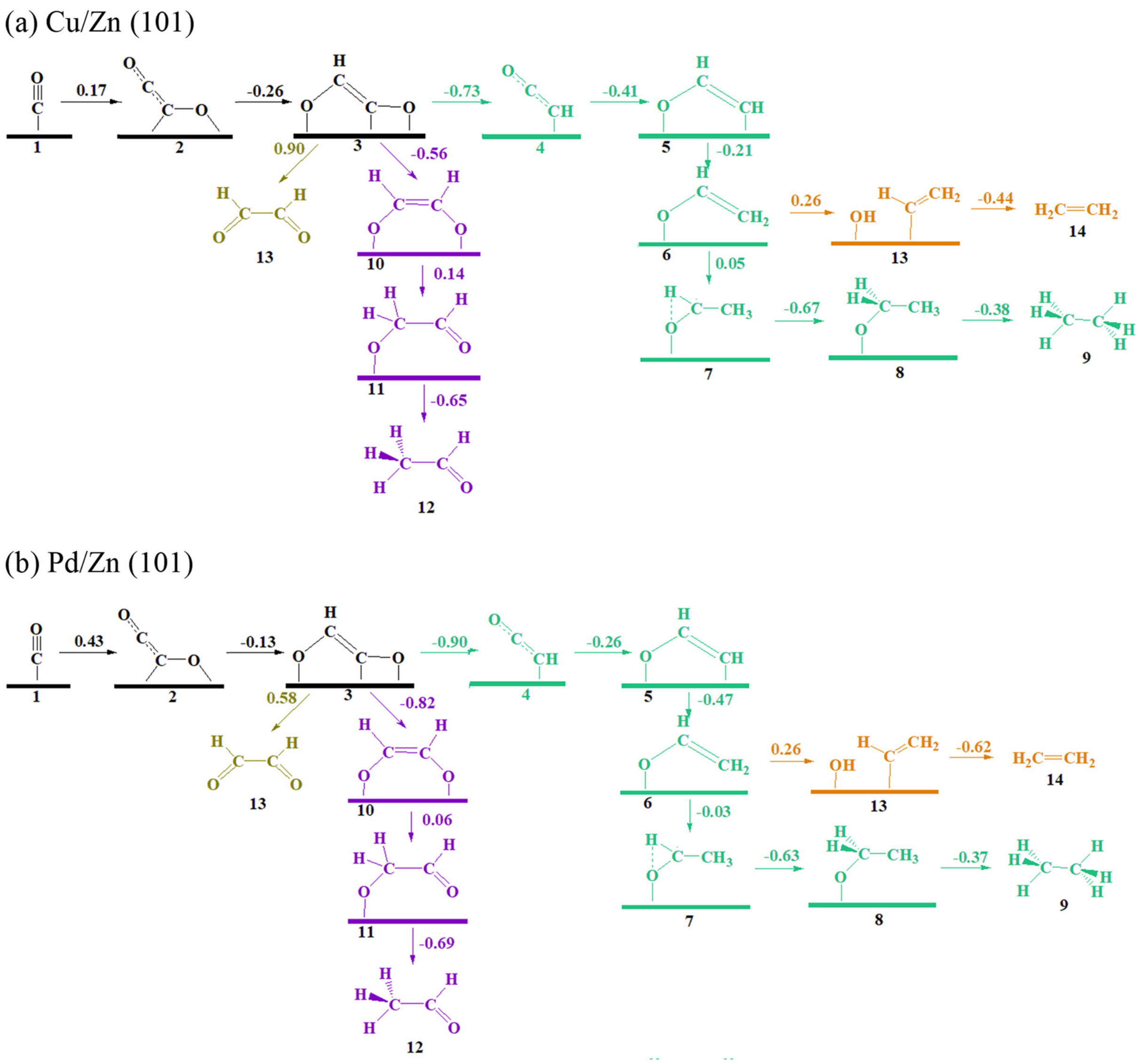

Figure S4. Mechanism of CO Reduction to C2 Products on Cu/Zn (101) (a) and Pd/Zn (101) (b). The black pathway represents the CO reduction reaction generating *CHOCO mechanism, the chartreuse pathway represents the acetaldehyde mechanism, the purple pathway represents the ethanol mechanism, the green pathway represents the methane mechanism, and the orange pathway represents the ethylene mechanism. In this figure, the free energy is indicated by the label near the arrow. The unit of measurement used is eV, and no voltage has been applied (0 V vs. RHE). As per convention, a large negative potential is considered to be a high potential.

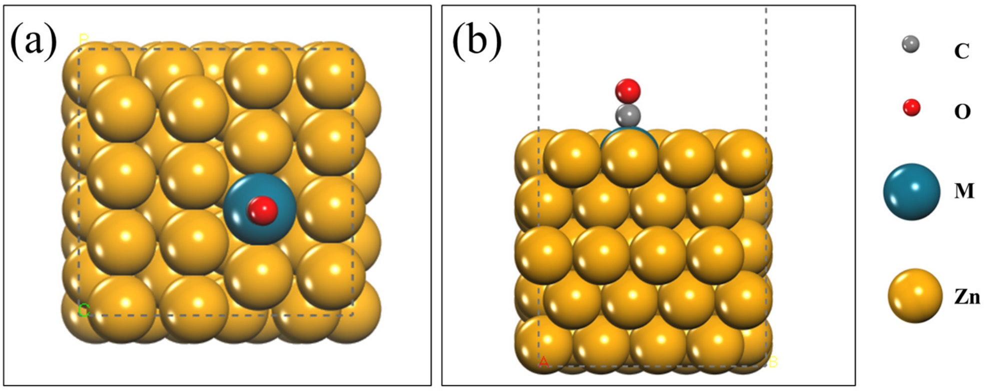

Figure S5. Structure *CO on M/Zn (101) surface, (a) top view, (b) side view.

Table S1. The potential required for the seven Zn SAAs to remain stable at common pH values in the reaction of M/Zn + H_2O → OH–M/Zn + H^+ + e^-.

M/Zn \ pH	Ag/Zn	Au/Zn	Cu/Zn	Ir/Zn	Pd/Zn	Pt/Zn	Rh/Zn
**0(vaccum)**	0.36	0.54	0.22	0.60	0.50	0.64	0.53
**5.6 (CO_2 - saturated electrolyte)**	0.03	0.21	−0.11	0.27	0.17	0.31	0.20
**6.8 (0.1 M $KHCO_3$ electrolyte)**	−0.04	0.14	−0.18	0.20	0.10	0.24	0.13
**7.0 (0.5 M KCl electrolyte)**	−0.05	0.13	−0.19	0.19	0.08	0.23	0.12
**7.2 (0.5 M $KHCO_3$ electrolyte)**	−0.06	0.12	−0.20	0.17	0.07	0.22	0.11
